# Use of the consolidated framework for implementation research in a mixed methods evaluation of the EQUIPPED medication safety program in four academic health system emergency departments

**DOI:** 10.3389/frhs.2022.1053489

**Published:** 2022-12-08

**Authors:** Michelle C. Kegler, Shaheen Rana, Ann E. Vandenberg, S. Nicole Hastings, Ula Hwang, Stephanie A. Eucker, Camille P. Vaughan

**Affiliations:** ^1^Rollins School of Public Health, Emory University, Atlanta, GA, United States; ^2^School of Medicine, Winship Cancer Institute, Emory University, Atlanta, GA, United States; ^3^Emory University School of Medicine, Atlanta, GA, United States; ^4^Duke University School of Medicine, Durham, NC, United States; ^5^Yale University School of Medicine, New Haven, CT, United States

**Keywords:** implementation science, Consolidated Framework for Implementation Research, mixed methods, emergency medicine, medication safety, older adults

## Abstract

**Background:**

Enhancing Quality of Prescribing Practices for Older Adults Discharged from the Emergency Department (EQUIPPED) is an effective quality improvement program initially designed in the Veterans Administration (VA) health care system to reduce potentially inappropriate medication prescribing for adults aged 65 years and older. This study examined factors that influence implementation of EQUIPPED in EDs from four distinct, non-VA academic health systems using a convergent mixed methods design that operationalized the Consolidated Framework for Implementation Research (CFIR). Fidelity of delivery served as the primary implementation outcome.

**Materials and methods:**

Four EDs implemented EQUIPPED sequentially from 2017 to 2021. Using program records, we scored each ED on a 12-point fidelity index calculated by adding the scores (1–3) for each of four components of the EQUIPPED program: provider receipt of didactic education, one-on-one academic detailing, monthly provider feedback reports, and use of order sets. We comparatively analyzed qualitative data from focus groups with each of the four implementation teams (*n* = 22) and data from CFIR-based surveys of ED providers (108/234, response rate of 46.2%) to identify CFIR constructs that distinguished EDs with higher vs. lower levels of implementation.

**Results:**

Overall, three sites demonstrated higher levels of implementation (scoring 8–9 of 12) and one ED exhibited a lower level (scoring 5 of 12). Two constructs distinguished between levels of implementation as measured through both quantitative and qualitative approaches: patient needs and resources, and organizational culture. Implementation climate distinguished level of implementation in the qualitative analysis only. Networks and communication, and leadership engagement distinguished level of implementation in the quantitative analysis only.

**Discussion:**

Using CFIR, we demonstrate how a range of factors influence a critical implementation outcome and build an evidence-based approach on how to prime an organizational setting, such as an academic health system ED, for successful implementation.

**Conclusion:**

This study provides insights into implementation of evidence-informed programs targeting medication safety in ED settings and serves as a potential model for how to integrate theory-based qualitative and quantitative methods in implementation studies.

## Introduction

Older adults are a vulnerable population at high risk for adverse drug events (ADEs), especially when they are discharged from the Emergency Department (ED) with a newly prescribed medication. Prescribing new medications for older patients outside the primary care setting increases the chances for suboptimal prescribing as well as ADEs, which are both major reasons for repeat ED visits, hospitalization and death (1–8). Recent studies show that more than half of older adults discharged from the ED leave with a new prescription medication (1, 2), and that the risk of it being a potentially inappropriate medication (PIM), one which could cause an ADE due to pharmacotherapy properties, physiological changes in aging, or limited efficacy in older adults, ranges from 5.6 to 13% (2–7). Prescribing safety and medication use among older adults is a public health concern and an important component of the “Medication” focus of the Age-Friendly Health System initiative (9, 10).

EQUIPPED (Enhancing Quality of Prescribing Practices for Older Adults Discharged from the Emergency Department) is an innovative quality improvement initiative designed to reduce PIM prescribing for adults aged 65 years and older (11). EQUIPPED comprises three intervention components: (1) provider education; (2) electronic health record (EHR) clinical decision support *via* specialized geriatric pharmacy order sets and links to online educational content at the point of prescribing; and (3) monthly provider feedback reports that include audit, feedback and peer benchmarking coupled with one-on-one provider academic detailing. EQUIPPED is informed by the Beers Criteria (12), evidence-based recommendations issued by the American Geriatrics Society that are widely used by government agencies and supported by research in various settings (1, 3, 5), to define PIMS and as a marker of prescribing quality in older adults. EQUIPPED is among a group of clinical decision support interventions that have been shown to be effective in changing provider behavior (13, 14). EQUIPPED has been successfully implemented in 20 urban and rural Veterans Affairs (VA) Medical Centers, with the first eight sites reducing PIM rates from a pre-implementation baseline of 7.4–11.9% of all prescriptions to 4.5–9.0% of prescriptions for 2 to 4 years after the initial 12-month implementation (11, 15) and additional results forthcoming. Early results based upon export and expansion of this VA innovation to additional VA and non-VA health systems also demonstrate reduction in PIMs at multiple sites (16–19).

Given the rapidity with which evidence is generated around safety and care of older adults and the often-cited timeframe of 17 years to move this evidence into practice (20, 21), it is vitally important to identify factors that facilitate more efficient and successful implementation and dissemination of evidence-informed interventions such as EQUIPPED into real-world settings. While a number of studies have examined outcomes associated with efforts to decrease PIMs in older adults (22–25), very few have evaluated the process of implementing evidence-informed interventions in EDs (26). Evaluating the implementation process intentionally and systematically using a theory-based approach will build the evidence-base for best practices such as EQUIPPED, and more generally, for common types of interventions such as provider education, clinical decision support, and academic detailing combined with audit, feedback and peer benchmarking across a range of settings and topics. In addition to medication safety for older adults, these strategies are commonly used to promote implementation of a broad range of clinical care guidelines, including for antibiotic prescribing, cancer screening, and mental health care, among other topics (27–29), with applicability in low, middle and high income countries (LMIC) (30, 31).

The Consolidated Framework for Implementation Research (CFIR) (32) is a widely used framework in implementation science designed to synthesize constructs from a range of theories and models (32, 33). It organizes 39 constructs and sub-constructs across five major domains and its consistent use across studies can help to build an evidence-base for factors that influence effective implementation. The majority of studies using CFIR have been qualitative, focusing on categorizing barriers and facilitators to implementation into CFIR domains and/or constructs (34–40). Relatively few studies have examined CFIR constructs quantitatively, in part because until recently there were few valid and reliable measures that clearly corresponded to CFIR constructs (41, 42). Moreover, given the many theories, models, potential measures of related constructs, overlapping definitions of similar constructs, and possible units of analysis, selection of appropriate measures for a specific intervention remains challenging (41, 42). Mixed methods studies of implementation, which capitalize on the strengths of both qualitative and quantitative approaches, have historically used quantitative methods to evaluate outcomes and qualitative approaches to document CFIR constructs related to implementation (43, 44). However, with more recently validated survey measures of CFIR constructs, there is now the opportunity to apply a fully mixed methods approach to understanding facilitators and barriers to implementation outcomes, such as fidelity of intervention delivery to a provider or patient population (45).

The purpose of the current study was to examine CFIR factors that influenced implementation of EQUIPPED in four non-VA, academic EDs from four distinct academic health systems using a mixed methods approach. In addition to providing insights valuable to implementing evidence-informed interventions for older adults in ED settings, this study serves as a potential model for how to integrate theory-based qualitative and quantitative methods in implementation studies.

## Methods

This study uses a convergent mixed methods design (46) that includes surveys of ED providers, focus group discussions with implementation team members, and program records as the data source for a measure of fidelity. Fidelity is defined as “the degree to which an intervention was implemented as it was prescribed in the original protocol or as it was intended by the program deliverer, p. 69.” (47).

Four EDs from four different academic health systems were purposively selected to extend implementation of EQUIPPED to new ED settings and different EHR platforms outside of the VA system where it was originally developed and tested. Three of the health systems use EPIC as the EHR platform, and these sites implemented EQUIPPED sequentially in successive years (2016–2019) under one funding mechanism. The fourth site uses Cerner and implemented EQUIPPED under a subsequent funding mechanism (2019–2021). Each of the selected sites included a clinical investigator who had been involved with the original evaluation of EQUIPPED in the VA system and who was affiliated with the corresponding academic health system. Each site PI formed a local implementation team that represented the skills needed to implement EQUIPPED, including at least one physician champion in the ED who was also a co-investigator on the research team. Implementation team members varied across sites but typically included geriatricians, ED physicians, pharmacists, EHR/IT experts, and a project coordinator. Implementation was sequential, one occurring each year, across the four sites (see Table 1), building program knowledge over time that could be applied at each subsequent site (48). PIMS-related outcomes for the first three EDs are reported elsewhere (16). The implementation evaluation study protocol was approved by the Emory University Institutional Review Board (IRB00087137).

**Table 1:** Emergency departments characteristics, selected implementation outcomes, and provider survey respondents.

**Descriptor**	**Site A**	**Site B**	**Site C**	**Site D**
**ED characteristics**				
Complexity	Level 1 trauma center	Level 3 trauma center	Level 1 trauma center	Level 1 trauma center
Patient Size (unduplicated)	112,446	88,968	53,324	33,856
Proportion patient population age 65%	15%	19.1%	21.2%	27%
Staff providers Percent attending physician	96 (55.2%)	60 (53.3%)	52 (73.0%)	65 (50.8%)
**Implementation (Fidelity) by component**				
Education Session ^a^ (estimated attendance)	Medium-2 66% attendance	Medium - 2 55% attendance	Medium-2 59% attendance	Low-1 20% attendance
Order Set Use ^a^ (% of discharge prescriptions based on one audit)	Low-1 1.2%	Low - 1 0.4%	Low-1 3.4%	Low-1 6.6%
Provider Feedback Reports	High-3 Went out monthly, but for those with PIMS only. Others received monthly congratulation e-mails.	Low-1 All got initial report, then quarterly for those with PIMs only.	High-3 Went out monthly to all	High-3 Went out monthly to all
One-on-one academic detailing ^a^	Medium-2 73%	Low-1 50% physicians and < 50% physician assistants	High-3 100%	High-3 96%
Fidelity score	8	5	9	8
Implementation order	1st	2nd	3rd	4th
**Provider survey respondents**				
**Profession**, ***n*****, %**				
Physician	25 (69.4%)	19 (65.5%)	19 (90.5%)	12 (70.6)
Nurse practitioner	8 (22.2%)	0 (0%)	0 (0%)	3 (17.7%)
Physician assistant	3 (8.3%)	10 (34.5%)	2 (9.5%)	2 (11.8%)
Male sex, *n*, %	16 (45.7%)	18 (64.3%)	11 (52.4%)	8 (47.1%)
Years at ED, mean, SD	10.7 (8.63)	9.3 (6.98)	9.2 (9.62)	9.5 (7.52)

a [High (≥80%), Medium (50–79%), Low (< 50%)].

### Data collection

#### Focus group discussions with implementation teams

The goal of the focus groups was to understand the facilitators and challenges faced in adapting and implementing EQUIPPED in the ED. Focus group discussions were conducted with each individual site at least 6 months after project initiation and after the last program components had been implemented, i.e., ED provider feedback reports distributed and one-on-one academic detailing provided. As mentioned, sites implemented EQUIPPED sequentially, with Site A implementing first in 2017 and Site D implementing last in 2020. All implementation team members at each site were invited to participate in the focus groups. E-mail invitations were sent by the site PI and/or research staff. The participation rate was 59.9% with variation across sites from 33.3 to 87.5%. The first three focus groups were conducted in-person and the last was conducted through ZOOM because of the COVID-19 pandemic. The number of participants from each site varied from 4 to 7, for a total *n* = 22 participants. Written informed consent was obtained from all participants. Each focus group discussion lasted approximately 90 min and was audio-recorded. All participants were compensated $30 for their time.

#### Provider surveys

The provider survey was administered following distribution of three provider feedback reports, and after one-on-one academic detailing was completed with the majority (>75%) of providers. All ED providers (i.e., attending physicians, nurse practitioners and physician assistants) were invited to complete a web-based survey about the implementation of EQUIPPED. An introductory e-mail was sent to providers to inform them of the survey, followed by a personalized link to a web-based survey programmed in REDCap. Up to five weekly reminders were sent. Overall, 108 of 234 providers completed the survey (response rate of 46.2%), with site-specific response rates ranging from 43.2 to 48.3%. Providers were compensated $20 for their participation.

#### Program records

Meeting minutes from both local sites and cross-implementation meetings were collected by the research team throughout the project. Implementation records on education (i.e., attendance records) and provider feedback (i.e., delivery logs) were requested from the local ED physician champion. Each individual site implemented its own audit of order set use and we included this as an indicator of fidelity of order set implementation, even though generation of a discharge prescription through use of the order set is not required for the intervention to be effective.

### Measures

#### Focus group discussion guide

The focus group discussion guide was designed to assess selected constructs within the CFIR domains of outer setting, inner setting, characteristics of the intervention, and the implementation process. As recommended by Damschroder et al. (32), a subset of 18 constructs was selected for this study, based on those that were potentially changeable and important (32, 41, 42). Specific focus group questions are listed by CFIR construct in Additional Files 1 and 2 and were adapted from prior qualitative research on evidence-based interventions to promote cancer screening and guidance from CFIR developers (https://cfirguide.org).

#### Provider survey questions

The provider survey similarly assessed constructs within the CFIR domains. The survey was largely adapted from validated measures and tailored for the ED setting with input from the study team (41, 42). Additional File 1 includes brief definitions, the number of items and sample questions for each construct assessed through the survey. Briefly, within the intervention characteristics domain, we assessed complexity (42, 49), and relative advantage (42, 50). For outer setting, we assessed external policies and incentives (42, 51), and patient needs and resources (42, 52). We assessed 11 constructs from the inner setting, including networks and communication (53, 54), two dimensions of culture (stress and effort) (41, 55, 56), implementation climate (41, 57), tension for change, relative priority (58), goals and feedback (42, 59), learning climate (41, 53), compatibility (42, 49), leadership engagement (41, 53, 60), available resources (41, 59), and access to information and knowledge (53). Within the process of implementation, we assessed engaging through champions (42) and reflecting and evaluating (42, 54). Response options varied from Yes/No to a 5-point Likert scale (1 = strongly disagree to 5 = strongly agree).

#### Implementation outcome: Fidelity

We used program records, including provider attendance records, audit results, meeting minutes, and program delivery logs, to create a measure of fidelity of delivering the intervention to the provider population. The four key intervention components were assessed for each site as follows: (1) education of providers was documented through attendance records (i.e., percentage of providers attending the session), (2) order set usage was assessed through clinical data warehouse extracts (i.e., percentage of prescriptions for older adults made through order sets during an audit period), (3) provider feedback (i.e., monthly to all providers or not) and (4) provider one-on-one education (i.e., percentage of providers meeting one-on-one with champion). Three of the components were each scored from 1 to 3 based on high, medium or low fidelity as follows: 3 = high (≥80%), 2 = medium (50–79%), 1= low (< 50%). Provider feedback was scored as 3 = High (monthly reports or congratulations e-mails to all providers), 2 = Medium (quarterly reports to providers), 1 = Low (quarterly reports to providers with PIMS only). An overall implementation fidelity score was then created by summing component scores, resulting in a possible range of 4 to 12. Once calculated, the scores were presented to site leads to confirm and validate the scoring and relative ranking.

### Data analysis

#### Qualitative analysis

Focus group discussions were recorded and transcribed verbatim. The initial codebook was based on the theoretical domains of CFIR. The codebook and code definitions were refined through coding of the first two transcripts, with additional codes added to capture emergent themes. All transcripts were coded independently by two analysts, with discrepancies resolved through discussion. NVivo 11 (QSR International) was used for data management and analysis.

NVivo reports were generated for each CFIR domain and construct, and these were used to prepare site-specific case studies. One analyst prepared all four case studies. The structure of the case studies was as follows: ED characteristics, implementation data from the provider survey and program records, and then five domain-specific sections. Each domain-specific section had scale scores and standard deviations for each construct from the provider survey, followed by a summary of qualitative findings for each construct. Additional analysis was then conducted using an approach similar to that of Damschroder et al. (61) and Liang et al. (35). Each construct was coded for valence, or the direction of each construct's influence on implementation, as expressed by the implementation team members at each site. Constructs were coded as positive (+), neutral (0), or a negative(-) influence on implementation, or not discussed (ND) per the approach described by Damschroder et al. (61). One analyst completed the initial assessment, with a second analyst reviewing the valence scores and disagreements resolved through discussion. The second analyst had visited three of the sites (not the fourth due to COVID), moderated the focus groups, and carefully reviewed the transcripts. A construct was rated as positive if it was described as a positive influence in the organization or a facilitating influence on work processes and/or implementation efforts. A construct was rated negatively if it was described as a negative influence in the organization or an inhibiting influence on work processes and/or implementation efforts (35, 61–63). A construct was rated neutral if there was no description of either a positive or negative influence and/or if descriptions were both positive and negative. Primary findings for each domain and construct were then placed into matrices ordered by level of implementation (i.e., fidelity) for cross case analysis and pattern identification (i.e., whether valence of a construct varied with the fidelity score).

#### Provider survey analysis

**Figure 1:**
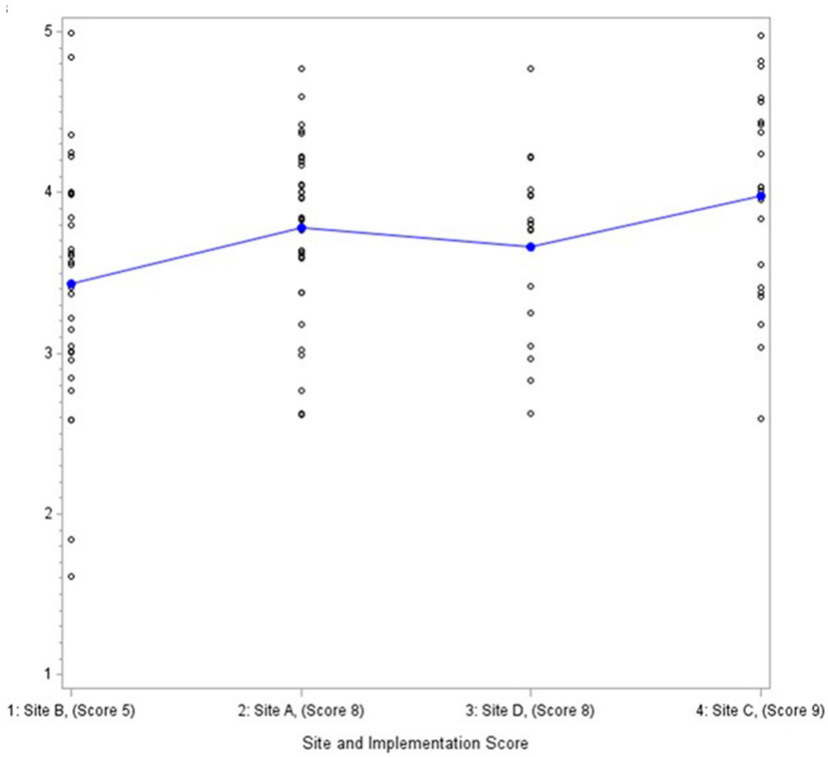
Communication and networks mean scores and distribution by site.

Data from the provider survey were analyzed descriptively with means, standard deviations and differences across sites calculated for each CFIR construct using the Statistical Analysis System (SAS) 9.4. Scales were formed by summing relevant items and then dividing by the number of items to create a scale score. Cronbach alphas were calculated on scales with three or more items to assess inter-item reliability. We examined differences in CFIR constructs across the four EDs using a ANOVA and Wilcoxon rank sum test. Given we were interested in organizational-level variables and the study had just four EDs, we then used graphical displays with sites ordered by fidelity score (Figure 1) to identify patterns associated with implementation fidelity descriptively. Consistent with a convergent mixed methods design, findings were then compared and contrasted across methods.

## Results

### Description of EDs and study participants

All four EDs were affiliated with academic health systems, and three of the four EDs were Level 1 trauma centers (Table 1). Numbers of staff providers per site ranged from 52 to 96; number of annual visits from unduplicated patients ranged from approximately 34,000 to 112,500. Proportion of the ED patient population comprising adults ages 65 years and older ranged from 15 to 27%. Table 1 also describes the survey respondents. Across all four EDs, 72.6% of survey respondents were physicians, 10.7% were nurse practitioners, and 16.5% were physicians assistants. The average tenure in the ED was 9.8 years (SD = 8.16) and across all EDs, 52.5% were men.

### Level of fidelity of delivery to provider population

Table 1 also shows fidelity of each intervention component as well as an overall fidelity score. Three of the four EDs had medium levels of provider attendance at the education sessions (range of 55 to 66%), with one ED reporting low attendance. Use of order sets to order medication prescriptions at discharge was low across all four EDs, ranging from 0.4 to 6.6% of all discharge prescriptions for older adults during the period audited. However, use varies by definition; a prior EQUIPPED evaluation indicated that 70% of providers used EQUIPPED order sets when use included consultation of the order sets as needed for a specific medication recommendation (64, 65). Provider feedback reports were categorized as high in three EDs, meaning that reports went out monthly to providers per the intervention design. The ED categorized as low for this component sent out an initial report, but then switched to quarterly distribution. Finally, two sites were classified as having high levels of delivery fidelity for the one-on-one academic detailing component, meeting with almost all of the providers at least once per the intervention design. One ED completed 73% of the one-on-one feedback sessions; and another was able to meet with 50% of their attending physicians and < 50% of the physician assistants. Overall, three sites demonstrated higher levels of implementation as operationalized through fidelity (Site A = 8, Site C = 9, and Site D = 8), with one ED exhibiting a lower level (Site B = 5).

### Findings by CFIR domain

In keeping with a mixed methods convergent design (46), qualitative and quantitative were first analyzed separately. Findings from the qualitative analysis are summarized in Table 2, with ED sites listed by level of implementation and each CFIR construct categorized as having a positive, negative or mixed influence on implementation within and across the EDs. Constructs that distinguished between high and low levels of implementation are also indicated. Table 3 presents the quantitative results in a site-ordered matrix with mean scores and standard deviation for each construct within each ED, significant differences between the EDs, and Cronbach's alpha when relevant. Table 4 synthesizes the qualitative and quantitative findings, which are discussed in detail below.

**Table 2:** Valence of CFIR constructs by domain as assessed through focus groups with implementation teams, site-ordered by level of implementation.

**Construct**	**Site C**	**Site A**	**Site D**	**Site B**	**Summary valence**
Implementation score	9	8	8	5	NA
**Intervention characteristics**					
Evidence strength and quality	+	+	+	+	Positive
Relative advantage	+	+	0	+	Positive
Adaptability	+	+	+	+	Positive
Trialability	0	0	+	+	Mixed
Cost	+	0	+	+	Positive
**Outer setting**					
Patient needs and resources	+	+	+	0	Distinguishing
External policies and incentives	+	+	+	+	Positive
**Inner setting**					
Structural characteristics	-	Not discussed	+	-	Mixed
Networks and communications	+	+	+	+	Positive
Culture	+	+	+	0	Distinguishing
Implementation climate	+	+	+	0	Distinguishing
Tension for change	-	0	0	-	Mixed
Compatibility	+	+	+	+	Positive
Relative priority	0	+	+	-	Mixed
Organizational incentives/rewards	+	0	+	0	Mixed
Readiness for implementation					
Leadership engagement	0	+	+	-	Mixed
Available resources	0	Not discussed	+	+	Mixed
Access to knowledge and information	+	+	+	+	Positive
**Process**					
External change agents	+	+	+	+	Positive
Formally appointed implementation team leaders	+	+	+	+	Positive
Champions	+	+	+	+	Positive

+ refers to positive, 0 refers to neutral, - refers to negative valence, or influence on implementation.

**Table 3:** Mean scores of CFIR constructs from ED provider survey by site level of implementation.

**Domain and construct**	**Site C**	**Site A**	**Site D**	**Site B**	***p*-value**	**Cronbach's alpha**
**Intervention characteristics**						
Relative advantage	3.8 (0.81)	3.8 (0.76)	4.0 (0.61)	3.8 (0.98)	0.80	NA
Complexity	2.1 (0.55)	2.3 (0.50)	2.4 (0.55)	2.2 (0.55)	0.26	0.74
**Outer setting**						
Patient needs/resources	4.1 (0.43)	3.7 (0.56)	3.7 (0.64)	3.6 (0.76)	0.01	0.73
External policies/incentives^*^	0 (0)	0 (0)	0.2 (0.71)	0.2 (0.37)	0.12^a^	NA
**Inner setting**						
Networks/communication	4.0 (0.63)	3.8 (0.52)	3.7 (0.58)	3.4 (0.78)	0.02	0.80
Culture-stress	3.4 (0.65)	3.9 (0.86)	3.5 (0.69)	4.4 (0.88)	0.0001	0.92
Culture-effort	4.2 (0.46)	4.1 (0.52)	3.7 (0.49)	3.9 (0.67)	0.03	0.74
Implementation climate	3.7 (0.42)	3.4 (0.58)	3.9 (0.38)	3.5 (0.77)	0.01	0.64
Tension for change	3.6 (0.73)	3.7 (0.67)	3.7 (0.85)	3.4 (0.74)	0.57	NA
Compatibility	4.0 (0.52)	3.7 (0.53)	3.9 (0.46)	3.6 (0.79)	0.09	NA
Relative priority	3.7 (0.41)	3.5 (0.57)	3.5 (0.54)	3.5 (0.63)	0.54	0.55
Goals/feedback	3.2 (0.82)	3.2 (0.75)	3.7 (0.79)	3.3 (0.77)	0.15	NA
Learning climate	4.3 (0.55)	4.2 (0.58)	3.9 (0.55)	3.9 (0.74)	0.04	0.86
**Readiness for implementation**						
Leadership engagement	4.2 (0.72)	4.0 (0.68)	3.9 (0.61)	3.3 (1.06)	0.001	0.93
Access to info/ knowledge	3.9 (0.54)	3.5 (0.65)	3.7 (0.47)	3.6 (0.77)	0.11	NA
Available resources	4 (0.51)	3.8 (0.38)	3.8 (0.57)	3.8 (0.67)	0.46	0.65
**Process**						
Champions	3.8 (0.64)	3.3 (0.6)	3.7 (0.58)	3.4 (0.76)	0.0331	NA
Reflecting and evaluating	3.5 (0.77)	2.9 (0.73)	3.8 (0.59)	3.5 (0.72)	< 0.0001	NA

Range 1 = strongly disagree to 5 = strongly agree for most items;

* Range 0 to 3;

a Wilcoxon rank sum test.

**Table 4:** Integrated results, CFIR construct influence on fidelity as an indicator of implementation quality.

**Construct**	**Qualitative**	**Quantitative**	**Distinguishing by both methods**
**Implementation score**			
**Intervention characteristics**			
Evidence strength and quality	Positive	–	–
Relative advantage	Positive	NS	No
Adaptability	Positive	–	–
Trialability	Mixed	–	–
Cost	Positive	–	–
Complexity	NA	NS	–
**Outer setting**			
Patient needs and resources	Distinguishing	Distinguishing	Yes
External policies and incentives	Positive	NS	No
**Inner setting**			
Structural characteristics	Mixed	Differs by Site	No
Networks and communications	Positive	Distinguishing	No
Culture	Distinguishing	Distinguishing (Stress)	Yes
Implementation climate	Distinguishing	Differs by Site	No
Tension for change	Mixed	NS	No
Compatibility	Positive	NS	No
Relative priority	Mixed	NS	No
Organizational incentives/rewards	Mixed	–	–
Goals and feedback	NA	NS	–
Learning climate	NA	Differs by Site	–
**Readiness for implementation**			
Leadership engagement	Mixed	Distinguishing	No
Available resources	Mixed	NS	No
Access to knowledge and information	Positive	NS	No
**Process**			
External change agents	Positive	–	–
Formally appointed implementation team leaders	Positive	Differs by site	No
Champions	Positive	Differs by site	No

NS, No significant differences by site; Not assessed (–), Differs by Site (statistically significant but does not correspond with level of implementation).

### Intervention characteristics

None of the intervention characteristics constructs differentiated levels of fidelity in delivery by ED (Tables 2, 3). The provider survey results on relative advantage showed no differences by ED, with respondents across all four EDs somewhat agreeing that EQUIPPED was better than their prior approach for decreasing PIMs (mean score of 3.8 to 4.0). In focus groups with the implementation teams, relative advantage was described as a positive influence in three of the four EDs. For example, in one site, team members stated they did not have anything “*systematic*” in place to address PIMS prior to EQUIPPED and would get “*gentle reminders from pharmacy saying maybe [they] shouldn't do that*.”

Qualitative and quantitative findings also aligned with respect to complexity of the intervention. Provider survey respondents somewhat disagreed with the perspective that EQUIPPED was very complex to implement (mean score of 2.1 to 2.4) (Table 3). Although not asked about explicitly in the focus groups, complexity of the intervention emerged in describing which components were easy or difficult to implement, largely in the context of building the order sets and navigating the approval process for changes to the EHR as opposed to the intervention itself being complex. Members from one team stated the order sets were the hardest to implement because “*there was a lot more red tape to get through and a lot more approval [they] had to get*.”

Of the additional constructs examined qualitatively within the intervention characteristics domain, evidence strength and quality and adaptability were viewed as positive influences in all four EDs (Table 2). For example, implementation team members commented that provider awareness of the Beers criteria, as well as knowledge the intervention was “*evidence-based medicine, that there's been previous…studies and literature that EQUIPPED has worked*” supported provider “*buy in*.” All of the sites described the intervention as adaptable and detailed specific adaptations to fit local needs and context, including changing the provider reports so they were more “*user friendly*,” tailoring the provider education presentation to an available faculty meeting time slot, and aligning the order sets with their work flows, EHR structure, and discharge procedures. These adaptations increased compatibility of the intervention within their settings. Cost was described as a positive influence on implementation in three of the four EDs. One team member emphasized that EQUIPPED cost “*a fraction of the money*” of another one of their initiatives, and they felt it “*was much more impactful*” due to being “*much more… focused*.”

None of the Intervention Characteristic constructs negatively influenced implementation of the intervention, and trialability, or the ability and usefulness of pilot testing, was mixed, in that it was described as positive in some sites and not relevant in others.

### Outer setting

One construct within the outer setting domain distinguished level of fidelity as assessed both qualitatively and quantitatively. The EDs varied significantly in the quantitative patient needs and resources measure (mean score of 3.6 to 4.1) (Table 3) in a direction consistent with implementation in that the ED with the highest score on patient needs and resources also had the highest level of fidelity. Patient needs and resources similarly emerged as a distinguishing factor in the qualitative analysis, with the three EDs with higher levels of fidelity describing their older patient populations and associated needs to address PIMs in this group as a facilitator to implementation, while the site with a lower level of fidelity described this factor as neutral (Table 2). For example, in one of the higher implementation EDs, team members indicated their ED has a “*large geriatric population*” and therefore EQUIPPED was a “*unique and great project for* [their] *ED*.” In contrast, one team member in the ED with lower fidelity described how EQUIPPED aligned well with the site's aging population, but that leadership did not recognize those needs or prioritize “*anything geriatric*.”

There were no significant differences in external policies/incentives across EDs on the provider survey, with all four very low on this measure (mean score of 0 to 0.2). Members of the implementation teams described external policies and incentives as a positive influence on implementation, particularly when aligning with quality measures. Some spoke broadly about how the program satisfied several certification and accreditation criteria and aligned with an increased government emphasis on quality, while others spoke specifically about working toward Geriatric ED certification and how EQUIPPED “*was an easy next step to try to move in that direction*.”

### Inner setting

Although operationalized differently, culture distinguished EDs by level of implementation in both the qualitative and quantitative data. Culture varied significantly across EDs from the provider perspective (mean scores of 3.4 to 4.4 for stress and 3.7 to 4.2 for effort), in a direction consistent with level of fidelity for the stress indicator (e.g., site with lower fidelity had higher stress). In the focus groups with implementation teams, a common theme across sites was a culture of research and quality improvement due to having academic faculty as providers and the teaching hospital culture. One participant described a “*good culture*” at their hospital and “*people are receptive to learning, especially if it's evidence based, since [they] are a teaching hospital*.” The site with the lower level of implementation, however, also described shifting priorities, changing leadership, and challenges with overcrowding and delayed hospital admissions. Although each of these fits within other domains, collectively they suggest a more turbulent culture than the other EDs.

Two additional inner setting constructs showed patterns consistent with the level of fidelity as assessed through the provider survey. The higher implementing sites scored higher on networks and communication (Figure 1) (mean scores of 3.4 to 4). In the focus groups, all four implementation teams described networks and communications as facilitating implementation. In one ED, team members stated they “*have a great working relationship in* [their] *department*” with a “*high level of trust among the entire group…including…working with* [PI and study coordinator].” This cohesion makes everyone feel “*comfortable bringing up issues*” and asking questions when they are unsure about anything. Leadership engagement (mean scores of 3.3 to 4.2) similarly distinguished fidelity as assessed through the provider survey, but was viewed as mixed in terms of positive or negative influence from the perspective of the implementation teams, and it did not distinguish higher from lower levels of fidelity.

One additional construct emerged as distinguishing from the qualitative data: implementation climate. Three of the EDs exhibited supportive implementation climates, albeit with different emphases. In one ED, the champion created excitement for the intervention, in others participation in a federally-funded research project helped to smooth implementation (e.g., paid time, higher visibility than a general quality improvement effort). In contrast, focus group participants at the ED with a lower level of fidelity described ED providers' initial concern that quality improvement or research projects might disrupt care; as EQUIPPED was minimally disruptive, it inspired little opposition but also little enthusiasm in this ED.

Compatibility and access to information were each positive influences on implementation in all four EDs and therefore not distinguishing based on the qualitative analysis (Table 2). In describing whether the intervention was compatible, one participant said, “*it's critically important that EQUIPPED was not designed to add time. If anything, it was to be neutral or reduce it, because with all of the pressures that EDs face*,” the intervention would not have been successful had it “*impede[d]*” their processes. With respect to accessing needed information, there was generally expertise on the team. As one participant stated, “*Identifying who needs to be on the bus, but that came pretty easily at this organization, and I think we got all the right people on the bus, so it made the process very smooth*.”

Several of the inner setting constructs were mixed in terms of their influence on implementation across the EDs, including structural characteristics, tension for change, relative priority, organizational incentives and rewards, leadership engagement as mentioned above, and available resources.

### Process of implementation

None of the constructs assessed within the process domain distinguished level of implementation. The provider survey assessed the constructs of champions, and reflecting and evaluating. Both varied significantly across sites (mean scores of 3.3 to 3.8 for champions, and 2.9 to 3.8 for reflecting and evaluating), but not in a pattern consistent with the level of fidelity.

Three of the constructs within the engaging domain were assessed for valence, and all were positive across all four EDs: implementation team leaders, champions and external change agents. Three of the EDs really highlighted their implementation team as using a shared leadership model, describing that while the PI led the implementation of EQUIPPED, they had “*a really distributed leadership model*” with the different team members taking responsibility for different aspects of implementation, depending on their expertise. ED physician champions were designated at the outset of implementation. For example, in one ED, participants spoke about how instrumental the champion was in keeping track of all the various components of EQUIPPED and making sure the project “*moved smoothly*,” as well as ensuring the ED was aware of the project and the various components, such as the order sets, so it would be viewed as a priority. The grant recipients (i.e., PI and team) were viewed as the external change agent, and their role was described favorably by all four sites. Evaluating and executing was discussed in terms of how the implementation team will assess whether the intervention was a success, rather than systems for ongoing monitoring and quality improvement.

## Discussion

Our mixed-methods analysis identified five CFIR constructs that distinguished the sites with the highest implementation of EQUIPPED from the site with the lowest implementation using fidelity as the implementation outcome of interest. Two constructs emerged consistently across both qualitative and quantitative data (*patient needs and resources* and *organizational culture*), one from the qualitative data only (*implementation climate*), and two from the quantitative data only (*networks and communication*, and *leadership engagement*). Additional factors positively influenced implementation across all four EDs as identified through the qualitative analysis, including: evidence strength and quality, relative advantage, adaptability, and cost from the intervention characteristics domain; external policies and incentives from the external setting domain; networks and communication, compatibility, access to information from the internal setting domain; and external change agent, appointment of a formal implementation team lead, and engagement of champions in the process domain.

Only one of the distinguishing factors was from a domain outside of the inner setting. The finding that *patient needs and resources*, an outer setting construct, was a distinguishing factor in implementation success suggests that EQUIPPED may be easiest to implement at sites which have, or are perceived to have, large geriatric populations with complex care needs that are known and prioritized. Such findings may transfer to analogous programs. Several other studies have similarly noted the salience of patient needs and resources in influencing implementation, sometimes as a distinguishing factor (61–63) and sometimes as salient barriers or facilitators to implementation (34, 35, 37, 39, 44). In environments such as the ED which see a diversity of patients and clinical presentations and have multiple competing priorities, patient subpopulation volumes may be important in driving organizational focus and support. This finding also points to the potential issue of ageism within health systems that may counter attempts to establish an Age-Friendly Health System (9).

The remaining distinguishing factors were from the inner setting domain. The finding that *organizational culture*, including lower stress and higher perceived work ethic, was associated with level of implementation suggests the importance of addressing cultural impediments before attempting to implement a new quality improvement program. For instance, timing of implementation should occur when space and attention can be devoted to it. The EQUIPPED site with the lowest fidelity of delivery reported many unforeseen changes during the period of implementation that may have limited team capacity for new program uptake. In contrast, it is also notable that one of the four sites was in the final stage of EQUIPPED implementation (initiating provider feedback) at the beginning of the COVID-19 pandemic. Despite this significant stressor for ED providers, the pandemic's impact only delayed completion of EQUIPPED implementation by a few months. For this site, the relative strength of the internal organizational culture may have mitigated the impact of a significant external challenge from derailing implementation. Organizational culture is a broad and multi-faceted construct as currently defined in CFIR which makes it challenging to compare findings across studies, with several reporting that it was not assessed or missing from qualitative data (35, 44, 61, 63) or not a distinguishing factor (62). We were able to identify a general “teaching” culture for all sites, with the fourth site also exhibiting a constellation of challenges which we coded as culture qualitatively as they aligned with the quantitative measures which focused on stress and effort.

Implementation climate is an overarching construct with several sub-constructs. Studies that have operationalized the sub-constructs and found some of them to distinguish levels of implementation are most common (35, 61–63). For example, Liang et al. observed that tension for change distinguished sites by level of implementation (35). Damschroder et al. (61) found that four of the sub-constructs distinguished level of implementation, including tension for change, relative priority, goals and feedback, and learning climate. We examined the sub-constructs, as well as an overall implementation climate characterized by overall receptivity to the intervention. Though *implementation climate* distinguished implementation level only qualitatively in our study, it suggests the need for implementation leaders and teams to closely attend to the degree to which its community members are receptive to quality improvement efforts such as EQUIPPED. Being attuned to stress and priorities within the organizational culture may also affect this climate and potentially shut down efforts to implement something new in the ED. The sites with the highest level of implementation were able to generate more enthusiasm among ED providers.

A large number of studies have identified that both networks and communication (61, 62) and leadership engagement (35, 61) are very important influences on implementation. Our study affirms that attention should be given to *networks and communication* and to *leadership engagement* as part of the implementation process, although identified only through the quantitative provider data. Those sites with higher perceived teamwork and regular communication among ED providers, and more engaged and supportive leaders, were able to implement the program more fully than the site with lower levels of these factors.

While we could not identify studies that specifically applied CFIR to understand implementation of medication safety programs for prescribers treating older adults in the ED, these findings may be considered in the context of other studies evaluating implementation of programs to influence prescribing behavior. A narrative review by Baumgartner et al. (26) highlighting factors abstracted from studies focused on de-prescribing inappropriate medications noted that *networks and* communication and patient needs and resources were important factors influencing implementation. Future research should examine whether different factors influence implementation based on setting (e.g., inpatient vs. outpatient), type of intervention (e.g., provider education, audit and feedback), implementation vs. de-implementation, or country context. For example, would culture, networks and communication, and leadership engagement still emerge as major influences on implementation of a medication safety program in LMIC, or would these factors be dwarfed by limited “available resources” in a low-income country? A recent review of CFIR use in over 30 LMIC countries reported general applicability across country context, along with recommendations for increased focus on characteristics of systems (e.g., systems architecture, resource continuity) (66).

### Limitations

This study has several limitations that should be considered when interpreting the results. In addition to a small number of EDs, we used just one implementation outcome for the comparative analysis: fidelity of delivery. Although the implementation science and CFIR literature is calling for more precise definitions and measurement of implementation (45), a more general measure of implementation outcomes may have led to different conclusions. Additionally, program records were used to determine delivery levels and these varied in quality. Focus groups did not include all members of the implementation teams and were therefore subject to the perspectives of those present. It is possible our finding would have differed if we had been able to include perspectives on implementation and CFIR constructs by role or position in the ED. Provider surveys too were a subsample of the entire provider sample and there could have been selection bias. Finally, this study did not examine whether increased fidelity or uptake of the intervention by providers was associated with improved PIMS outcomes. Despite these limitations, our data on implementation is representative for EQUIPPED based upon the balanced response rate across sites and the range of detailed data sources leveraged in this mixed methods analysis.

## Conclusion

Few studies have evaluated implementation factors for geriatric care programs in the ED setting (26). Our mixed methods analysis triangulates not only different data sources (surveys and focus groups) but also differing perspectives (the implementation team vs. ED providers). Organizational culture, the extent to which the needs of older patients are known and prioritized, strong networks and communication, and leader engagement emerged as particularly important in successful implementation of EQUIPPED. As the Age-Friendly Health System movement grows, programs like EQUIPPED provide clinical leaders in the ED with a blueprint for optimizing prescribing behavior toward older adults. Because there are few implementation studies of quality improvement programs in the ED focused on geriatric care, the current findings are an important first step toward advancing best practices to enhance health care delivery for older adults in the ED.

## Data availability statement

The datasets presented in this article are not readily available because one of the datasets generated and/or analyzed during the current study is not publicly available due to its qualitative nature and difficulty in making it non-identifiable. The survey data are available from the corresponding author on reasonable request. Requests to access the datasets should be directed to mkegler@emory.edu.

## Ethics statement

The studies involving human participants were reviewed and approved by Emory University Institutional Review Board. The patients/participants provided their written informed consent to participate in this study.

## Author contributions

MK designed the study and data collection instruments, drafted major sections of the manuscript, collected the qualitative data, analyzed qualitative and quantitative data, and edited the full manuscript. SR coordinated data collection, analyzed qualitative data, wrote parts of the results, and edited the full manuscript. AV helped design the study, drafted sections of the introduction and discussion, and edited the full manuscript. SH helped design the study, co-coordinated implementation of the intervention in one ED, and edited the full manuscript. UH helped design the study, coordinated implementation of the intervention in one ED, and edited the full manuscript. SE co-coordinated implementation of the intervention in one ED and edited the full manuscript. CV helped design the study, oversaw implementation of the intervention as overall PI, wrote part of the discussion section, and edited the full manuscript. All authors approved the final version of the paper.
