# Supplementary material for: Use of the consolidated framework for implementation research in a mixed methods evaluation of the EQUIPPED medication safety program in four academic health system emergency departments
Source: Front Health Serv. 2022 Dec 8;2:1053489. doi: 10.3389/frhs.2022.1053489 (PMC10012623; doi:10.3389/frhs.2022.1053489)
Supplement: Supplementary file 1 [file Table_1.DOCX]

**Additional Table 1. Description of Measures by Construct and Domain**

| **Domain, Construct, and Definition (from Damschroder et al., 2009)** | **Focus Group Questions & Key Probes** | **Provider Survey, Number of items, and Example Item** |
| --- | --- | --- |
| **Intervention Characteristics** |  |  |
| Evidence Strength/ Quality  *Stakeholders’ perceptions of the quality and validity of evidence supporting the belief that the*  *intervention will have desired outcomes* | How convincing was the evidence that EQUIPPED would work in your ED? | NA |
| Relative Advantage  *Stakeholders’ perception of the advantage of implementing the intervention versus an alternative solution.* | What did you have in place to prevent PIM prior to EQUIPPED? | 1 item  The EQUIPPED program is more effective than our prior practices for decreasing PIMs. |
| Adaptability  The degree to which an intervention can be adapted, tailored, refined, or reinvented to meet local needs. | How did you change EQUIPPED to fit your ED? | NA |
| Trialability  *The ability to test the intervention on a small scale in the organization [8], and to be able to reverse course (undo implementation) if warranted.* | Did you pilot test any aspect of EQUIPPED before rolling it out to your providers? | NA |
| Complexity  *Perceived difficulty of implementation, reflected by duration, scope, radicalness, disruptiveness,*  *centrality, and intricacy and number of steps required to implement.* | NA | 4 items  Overall, I believe that it is complicated to implement EQUIPPED. |
| Cost  *Costs of the intervention and costs associated with implementing that intervention including*  *investment, supply, and opportunity costs.* | Think about the money, time, and other costs that went into implementing EQUIPPED. How do those compare to implementation of other evidence-based interventions? |  |
| Not Assessed: Design Quality/ Packaging; Source |  |  |
| **Outer Setting** |  |  |
| Patient needs/resources  *The extent to which patient needs, as well as barriers and facilitators to meet those needs are*  *accurately known and prioritized by the organization.* | How well does EQUIPPED align with the needs of your patient population? | 3 items  This ED does a good job assessing patient needs and expectations. |
| External Policies/incentives  *A broad construct that includes external strategies to spread interventions including policy and regulations (governmental or other central entity), external mandates, recommendations and guidelines, pay-for-performance, collaboratives, and public or benchmark reporting.* | Please describe any external incentives in place to promote EQUIPPED. | 3 items  During the past academic year, did your ED receive any additional income or reimbursement for scoring well on PIM quality measures? |
| Not assessed: : Cosmopolitanism and Peer Pressure |  |  |
| **Inner Setting** |  |  |
| Structural Characteristics  *The social architecture, age, maturity, and size of an organization.* | In what ways did the structure of your ED help or hinder implementation of EQUIPPED? |  |
| Networks/Communication  *The nature and quality of webs of social networks and the nature and quality of formal and informal*  *communications within an organization.* | What methods did you use to communicate as a team and across the organization to get EQUIPPED implemented? How effective were they? | 5 items  Communication among leaders and staff in this ED is productive. |
| Culture  *Norms, values, and basic assumptions of a given organization.* | We’d like to know about how the culture of your ED may have influenced implementation of EQUIPPED. What comes to mind? | Stress (4 items)  Staff often show signs of stress and strain.  Effort (5 items)  Staff in this ED always want to perform to the best of their abilities. |
| Implementation Climate  *The absorptive capacity for change, shared receptivity of involved individuals to an intervention and the extent to which use of that intervention will be rewarded, supported, and expected within their organization.* |  | 3 items  ED providers are expected to help EQUIPPED meet its goal. |
| *Tension for change*  *The degree to which stakeholders perceive the current situation as intolerable or needing change.* | To what extent were people in your ED interested in addressing PIMs for older adults before you learned about EQUIPPED | 1 item  We needed to do something to better manage PIMS among older adults. |
| *Compatibility*  *The degree of tangible fit between meaning and values attached to the intervention by involved individuals, how those align with individuals’ own norms, values, and perceived risks and needs, and how the intervention fits with existing workflows and systems.* | How well does the intervention fit with existing work processes and practices in your setting? | 2 items  Using the EQUIPPED program to decrease PIM rates is compatible with current activities/practices in the ED. |
| *Relative priority*  *Individuals’ shared perception of the importance of the implementation within the organization.* | Compared to other priorities your ED may have, in general, how important is EQUIPPED? Are there competing priorities? | 3 items  Using the EQUIPPED program to decrease PIMS is a top priority of the ED. |
| *Organizational incentives/rewards*  *Extrinsic incentives such as goal-sharing awards, performance reviews, promotions, and raises in salary and less tangible incentives such as increased stature or respect.* | Please describe any internal incentives to support the implementation of EQUIPPED. | NA |
| *Goals/feedback*  *The degree to which goals are clearly communicated, acted upon, and fed back to staff and alignment of that feedback with goals.* |  | 2 items  ED leaders establish clear goals for EQUIPPED to decrease PIMs. |
| *Learning Climate*  *A climate in which: a) leaders express their own fallibility and need for team members’ assistance and input; b) team members feel that they are essential, valued, and knowledgeable partners in the change process; c) individuals feel psychologically safe to try new methods; and d) there is sufficient time and space for reflective thinking and evaluation.* |  | 5 items  We regularly take time to consider ways to improve how we do things. |
| Readiness for Implementation |  |  |
| *Leader engagement*  *Commitment, involvement, and accountability of leaders and managers with the implementation* | In what ways did your leadership help or hinder implementation of EQUIPPED? | 4 items  Leadership strongly supports ED change efforts. |
| *Available Resources*  *The level of resources dedicated for implementation and on-going operations including money, training, education, physical space, and time.* | Were adequate resources available to implement the intervention? Is there anything that you needed that you did not have access to? | 4 items  We have adequate resources available to implement this program. |
| *Access to info/ knowledge*  *Ease of access to digestible information and knowledge about the intervention and how to*  *incorporate it into work tasks.* | To what extent would you say your team had the knowledge and skills you needed to implement the various components of EQUIPPED? What additional skills would have been helpful? | 2 items  Providers in this ED have the information they need to use the EQUIPPED program well. |
| **Process** |  |  |
| Engaging |  |  |
| *Formally appointed internal implementation leaders*  *Individuals from within the organization who have been formally appointed with responsibility for implementing an intervention as coordinator, project manager, team leader, or other similar role.* | Other than you, who were the individuals involved in implementing EQUIPPED, and what was their role?  Who led day-to-day implementation of EQUIPPED? |  |
| *Champions*  *“Individuals who dedicate themselves to supporting, marketing, and ‘driving through’ an [implementation]” [101](p. 182), overcoming indifference or resistance that the intervention may provoke in an organization.* | Who served as champion(s)? What did they do in this role? | 2 items  Some of our staff have become program champions, actively supporting and promoting EQUIPPED beyond what is required. |
| *External Change Agents*  *Individuals who are affiliated with an outside entity who formally influence or facilitate intervention decisions in a desirable direction.* | What do you think about the communication from Emory? |  |
| Executing  *Carrying out or accomplishing the implementation according to plan.* | Which aspects of the intervention were easy to implement and why? |  |
| Reflecting and Evaluating  *Quantitative and qualitative feedback about the progress and quality of implementation with regular personal and team debriefing about progress and experience.* | How will you decide if EQUIPPED or its components are a success? | Throughout the ED there is frequent and good communication about how EQUIPPED is going.  We use data (e.g., performance reviews, assessments) to guide implementation of EQUIPPED. |
| Not Assessed: Planning, opinion leaders |  |  |
