# Supplementary material for: Use of the consolidated framework for implementation research in a mixed methods evaluation of the EQUIPPED medication safety program in four academic health system emergency departments
Source: Front Health Serv. 2022 Dec 8;2:1053489. doi: 10.3389/frhs.2022.1053489 (PMC10012623; doi:10.3389/frhs.2022.1053489)
Supplement: Supplementary file 2 [file Data_Sheet_1.docx]

**Enhancing Quality of Prescribing Practices for Older Adults**

**Discharged from the Emergency Department**

**(EQUIPPED)**

**Moderator Guide**

**Interviewer Self-Introduction (2 min)**

(Name, title, connection with project)

**Introduction of EQUIPPED (4 min)**

EQUIPPED stands for Enhancing Quality of Prescribing Practices for Older Adults

Discharged from the Emergency Department. It is a program to reduce potentially inappropriate medications (PIMs) prescribed to adults age 65 and older who are discharged from the ED. It has three core components: 1) provider education (didactic education, certification of ED pharmacists in Geriatric Pharmacy Practice Certification, and reminder cards), 2) electronic health record (EHR)-based clinical decision support, and 3) ongoing provider feedback through regular academic detailing and peer benchmarking. EQUIPPED was originally implemented at 8 Veterans Administration Medical Centers and now is being implemented at three non-VA hospitals. Yours is one of them. Today we would like to ask you about your experience with the EQUIPPED program so that we can learn about which aspects of implementation have worked and which could be improved.

**Informed- Consent and Conduct brief survey (5 min)**

Before we begin, I’d like to review a consent form with you that tells you more information about this discussion group.

(Moderator or nonverbal note-taker will distribute the consent document.)

This document explains the purpose of the discussion group and what you can expect while you’re here. Let’s go over the key points.

[Conduct Informed Consent process.]

**Guidelines**

Here are some rules for the discussion group:

- Please try to talk one at a time and take turns talking.
- We are very interested in your opinions. There are no right or wrong answers, only different ideas. We welcome your honest opinions.
- Also at this time please turn off cell phones, if you are able to do so.
- Are there any questions before we begin?

We are going to start recording now. (Moderator/Notetaker will start the audio recorder.)

**Introduction of the implementation team (5 min)**

(Go around circle and have everyone speak):

To start with, please let me know your first name, your job within the ED, and your role in the EQUIPPED project.

1. **Adoption**

Thank you. In a minute, I’m going to ask you about ***how*** you implemented EQUIPPED, but first I’d like to ask ***why*** your ED decided to implement EQUIPPED?

Probes:

How did you come to be interested in implementing EQUIPPED?

What did you have in place to prevent PIMs prior to EQUIPPED? What are the relative advantages of these two different approaches to preventing PIMs – EQUIPPED versus [prior approach]?

How well does EQUIPPED align with the needs of your patient population?

1. **Process of Implementation**

Now we’d like to hear about your organizational process for implementing EQUIPPED here at ________.

How did you introduce EQUIPPED to your ED? What steps were taken to plan for implementation?

(Display draft timeline of implementation on white board.)

Please review this draft timeline of implementation prepared from your meeting agendas—What, if anything, needs changing?

(Work collaboratively to make changes to it during discussion.)

Probes:

Other than you, who were the individuals involved in implementing EQUIPPED, and what was their role?

Who led day-to-day implementation of EQUIPPED?

Who served as champion(s)? What did they do in this role?

Which aspects of the intervention were easiest to implement and why?

Which aspects of EQUIPPED here hardest to implement and why? ***Probe on specific components as needed***: What about [education, order sets, provider feedback]?

Did you pilot test any aspect of EQUIPPED before rolling it out to your providers?

Please describe any external incentives that align with the goals of EQUIPPED. (These may be local, state, or national performance measures, policies, regulations, or guidelines.)

How will you decide if EQUIPPED or its components are a success?

1. **Intervention Characteristics**

We’d also like to know about personal reactions to the program. How have people in your ED reacted to EQUIPPED?

Probes:

How convincing was the evidence that EQUIPPED would work in your ED?

How did providers view EQUIPPED when it was first introduced?

Were there any groups of providers or staff that were resistant to implementing EQUIPPED?

How did you change EQUIPPED to fit your ED? [Education, order sets, provider feedback forms]

Think about the money, time, and other costs that went into implementing EQUIPPED. How do those compare to implementation of other evidence-based interventions

1. **Implementation Team Training and Capacity**

Another important aspect of implementation is human capital. To what extent would you say the implementation team had the knowledge and skills you needed to implement the various components of EQUIPPED?

Probes

What about education about geriatric prescribing?

Order sets development?

Provider feedback?

What additional skills would have been helpful?

Describe any technical assistance you got from Emory. Would you say the amount was too much, not enough, or about right?

What do you think about the communication from Emory?

How helpful were the Wednesday afternoon Vidyo calls?

How about the Monday all-site conference calls?

Any advice you’d like to give about how Emory could better support implementation of EQUIPPED?

1. **Inner Setting**

We’d like to know about how the culture of your ED may have influenced implementation of EQUIPPED. What comes to mind?

Probes

To what extent were people in your ED interested in addressing PIMs for older adults before you learned about EQUIPPED?

How does implementation of the intervention align with other organizational goals?

Compared to other priorities your ED may have, in general, how important is EQUIPPED? Are there competing priorities?

How well does the intervention fit with existing work processes and practices in your setting? Does it disrupt processes in any way?

Please describe any aspects of your ED culture that helped or hindered your efforts to implement EQUIPPED

Please describe any internal incentives to support the implementation of EQUIPPED.

In what ways did your leadership help or hinder implementation of EQUIPPED?

In what ways did the structure of your ED help or hinder implementation of EQUIPPED? Were adequate resources available to implement the intervention? Is there anything that you needed that you did not have access to?

What methods did you use to communicate as a team and across the organization to get EQUIPPED implemented? How effective were they?

What was the general level of receptivity in your ED to implement EQUIPPED?

1. **Maintenance**

To wrap up, which parts of EQUIPPED, if any, do you expect to continue after the current grant ends?

Probes

***Probe on specific components as needed:*** What about [provider education, order sets, provider feedback,]?

Why do you think that these will continue?

Please describe any steps you have already taken to make sure that these continue.

What resources do you need to maintain these parts of EQUIPPED?

Anything else you would like to share with me about implementation of EQUIPPED?

**Distribution of the gift cards**

(Moderator or nonverbal note-taker will distribute the $30 gift cards and accompanying receipt list.)

| NOTES: |
| --- |
